# The effects of nuts intake on cognitive and executive function in obese children: a randomized clinical trial

**DOI:** 10.1186/s41043-025-00804-7

**Published:** 2025-03-12

**Authors:** Fatemeh Sheikhi, Amir Bagheri, Reza Amani, Aliakbar Foroughi, Mehdi Moradinazar, Mehnoosh Samadi

**Affiliations:** 1 Nutritional Sciences Department, School of Nutrition Sciences and Food Technology, Kermanshah University of Medical Sciences, Kermanshah, Iran; 2 Clinical Nutrition Department of Clinical Nutrition School of Nutrition and Food Science Food Security Research Center, Isfahan University of Medical Sciences, Isfahan, Iran; 3 Department of Clinical Psychology, Kermanshah University of Medical Sciences, Kermanshah, Iran; 4 Behavioral Disease Research Center, Kermanshah University of Medical Sciences, Kermanshah, Iran; 5 Research Center for Environmental Determinants of Health (RCEDH), School of Public Health, Kermanshah University of Medical Sciences, Kermanshah, Iran

**Keywords:** Nuts, Obesity, Children, Cognitive function, Executive function

## Abstract

**Background:**

Childhood obesity is a significant, worldwide challenge disrupting various body organs including the brain. Studies suggest that nuts, rich in nutritional compounds, can improve cognitive function. This study aimed to investigate the effects of consuming nuts on improving cognitive and executive function in obese children.

**Methods:**

In this randomized parallel clinical trial, ninety 8 to 10-year-old girls with obesity were divided into intervention (30 g of nuts/daily) and control groups (no nuts) for 8 weeks. Furthermore, the Wechsler Four (WISC-IV) questionnaire assessed children’s cognitive function, and executive function was assessed by the behavior rating inventory of executive function (BRIEF) parental questionnaire. Linear mixed-effect models were done to survey the effects of nut intake on cognitive and executive function.

**Results:**

Forty people with a mean age of 9 ± 0.7 years from each group cooperated to the end of the study. The intervention group showed a significant improvement in the total Wechsler score (differences: 23.1, 95% CI: 17.5, 28.7; *P*_Time×Group_ < 0.001) and total BRIEF score (differences: − 16.5, 95% CI: − 29.4, − 3.5; *P*_Time×Group_ < 0.05) compared to the control group. Moreover, other components of the Wechsler and BRIEF tests including picture completion, picture concept, block design, object assembly, short-term memory, digit span, inhibition, displacement, initiation, and organization were significantly improved in the nuts group compared to the control group after the 8th weeks.

**Conclusion:**

These results underline the potential of nut consumption as a dietary intervention to improve cognitive function over an eight-week period, highlighting its role in supporting brain health and cognitive development of obese children.

**Supplementary Information:**

The online version contains supplementary material available at 10.1186/s41043-025-00804-7.

## Introduction

Childhood obesity is a major global challenge that has increased dramatically in recent decades [1]. It is estimated by 2030, 254 million children aged 5–19 worldwide will be obese [2]. The Covid-19 pandemic, school closures, reduced physical activity, and lifestyle changes have exacerbated obesity in children [3]. Obesity in children has adverse short- and long-term consequences. It causes metabolic and cardiovascular disorders and increases the risk of endocrine diseases and cancer [4]. Recent studies have shown that obesity and increased body fat mass in children affect the brain’s health, the structure of the brain, and cognitive and executive function [5–8]. Ultimately, obesity and cognitive dysfunction negatively affects the quality of life, and personal and general health [1, 9, 10]. It shows that obesity in children and adolescents is associated with a decrease in gray matter and white matter of the brain in areas related to cognitive control and learning compared to children and adolescents with a healthy weight [11]. In children with more central obesity, decreased ability in mathematics, reading, and spelling abilities were observed even after controlling the IQ. It has explicitly been shown that children with obesity have reduced executive function, attention span, short-term memory, and spatial-visual and verbal abilities [8]. Nuts have essential compounds for health, including beneficial fatty acid profiles that contain large amounts of polyunsaturated fatty acids (PUFAs) and monounsaturated fatty acids (MUFAs) and small amounts of saturated fatty acids (SAFAs). Nuts are also rich in fiber, minerals, phytochemicals, and antioxidants [12, 13]. Among them, walnut, almond, and hazelnut have unique ingredients in addition to those found in other nuts. Walnut is a rich source of omega-3 fatty acids, especially alpha-linolenic acid (ALA) [14]; almond is an excellent source of arginine, polyphenols, and antioxidants [15], and hazelnut is a rich source of magnesium, copper, and selenium [16]. It can protect and maintain proper functioning of the brain and prevent its deterioration. Nuts intake is inversely related to chronic diseases that cause mortality, such as cardiovascular disease, hypertension, metabolic syndrome, and diabetes [17–22], and can improve inflammatory markers, insulin sensitivity, and weight control [23–25].

In addition, cohort studies [26–28] and cross-sectional studies [29] have shown that long-term consumption of nuts is associated with better cognitive function in adults at different ages and can improve cognitive function or delay cognitive decline [30]. Also, it has been proposed that replacing solid snacks with tree nuts in children can enhance the diet quality to receive a rich source of micronutrients and macronutrients [31, 32]. Recent studies have found that children have a greater degree of neuroplasticity, and improving cognitive function in childhood can have better effects on brain function and prevent deterioration and cognitive decline later in life [33–36]. Investigating the effect of nuts consumption on the cognitive performance of children may provide insight into the mechanisms involved in any changes in neurocognitive performance. As a result, this study aimed to investigate the effect of consuming walnuts, almonds, and hazelnuts for eight weeks on the cognitive and executive functions of obese children.

## Materials and methods

This study was conducted as a randomized parallel clinical trial for 8 weeks from November 2019 to March 2020 at the schools of Kermanshah city (the west of Iran). The study protocol was approved by the Kermanshah University of Medical Sciences Ethics Committee (IR.KUMS.REC.1398.365) and was registered in the Iranian Clinical Trials Center at IRCT20181111041611N2 on September 2019, and available through www.irct.ir. By referring to the education department and accessing the list of schools in Kermanshah city, ninety students who met the study entry criteria were randomly selected. Initially, the study was fully explained to the children and their parents, and written informed consent was obtained from all of the participants.

### Participants

Ninety participants were matched based on age, Wechsler score (with a maximum of 15 points difference), and BMI (2 points difference). Both groups were randomly selected from the same catchment area. The initial study was defined in 16 weeks. However, due to the simultaneous onset of the Covid-19 pandemic and lockdown, all the tests were performed at the start (0 weeks) and the end (8 weeks) of the study period. Participants were randomly assigned to either the intervention or control group using a computer-generated random sequence to ensure an even probability of being placed in either group. The allocation sequence was placed in sealed, opaque envelopes, which were opened only after the participant had been enrolled in the trial. Additionally, we ensured that allocation concealment was maintained throughout the process. Inclusion criteria include at least three years of obesity, lack of underlying diseases such as diabetes, endocrine disease, cardiovascular and liver disease, not taking drugs that lead to obesity, not doing professional exercise, and not having an allergy to nuts. In addition, during the study, people who did not want to continue started exercising, dieting, became sick, or stopped eating nuts were excluded from the study [25, 37–39]. The intervention group received 30 g of nuts (walnuts 15 g, almonds 7.5 g, and raw Persian hazelnuts 7.5 g) in two 15 g doses (morning and evening) at home [38]. The control group did not receive any nuts. Telephone follow-ups in this regard were performed by the researchers every week (35). To weight gain prevention and control calorie intake regarding nuts, the parents of the intervention group were asked to reduce the amount of 150 kcal from the children’s carbohydrate and fat groups. Individuals were also asked not to change their diet and physical activity and during the study work. To evaluate the types and amounts of fatty acids and proteins in the used nuts, they were tested in the specialized Kimia Pajooh Alborz laboratory of food, minerals, and environment. The results are demonstrated in Supplemental Table 1.

**Table 1:** General characteristics of study participants

Variable		Control (N = 40) mean ± SD	Nuts (N = 40) mean ± SD	*P* value
Age (years)		9 ± 0.7	9 ± 0.7	1
Birth weight (g)		3227 ± 717.63	3234.25 ± 399.90	0.77
Birth rank		1.4 ± 0.64	1.5 ± 0.81	0.54
Watching TV (hours)		3 ± 2.5	3.5 ± 2.9	0.12
Computer games (hours)		1.1 ± 0.86	1 ± 0.76	0.90
Mother’s age (years)		37 ± 5.3	38 ± 6.2	0.42
Maternal delivery age (years)		28 ± 5.4	28.4 ± 6.2	0.59
Mother’s BMI (kg / m2)		29 ± 3.1	29.5 ± 4.8	0.42
Family member (persons)		4.1 ± 0.85	4.1 ± 0.88	1
Father’s education	Diploma and sub-diploma	20(45.6)	23(53.4)	0.462
	Post-diploma and bachelor’s degree	15(60)	10(40)	
	Masters and PhD	5(41.6)	7(58.3)	
Mother’s education	Diploma and sub-diploma	28(53.8)	24(46.1)	0.188
	Post-diploma and bachelor’s degree	12(48)	13(52)	
	Masters and PhD	0(0)	3(100)	
History of obesity	No	1(16.6)	5(83.3)	0.151
	Paternal family	10(66.6)	5(33.3)	
	Maternal family	9(60)	6(40)	
	Paternal and maternal family	20(45.4)	24(54.5)	
History of chronic diseases	No	10(58.8)	7(41.1)	0.412
	Yes	30(47.6)	33(52.5)	
Having breakfast	No	11(50)	11(50)	1
	Yes	29(50)	29(50)	

### Anthropometric information

The subjects’ height was measured with a Seca gauge without shoes and with an accuracy of 0.5. Weight, BMI, FMI, and body fat percentages were measured with a body analyzer (Jawon Medical Plus model Avis 333, Janex Medical Co., Seoul, Korea). We used BMI for the age percentile of the CDC standard growth chart and children with more than 95% BMI for the age percentile were defined as obese children. Physical activity was assessed through the Beck questionnaire for this age group [40].

### Nutritional assessment

The food intake status of participants was assessed by a three-day food registration questionnaire (including two non-holiday and one day off) at weeks 0 and 8, and then analyzed by nutrition IV software.

### Cognitive and executive function assessment

In this study, we used two tools to examine cognitive function. The Wechsler Four WISC-IV questionnaire assessed children’s cognitive function. It addresses the general functioning of intelligence, and verbal and nonverbal intelligence, as well as comprehension, perceptual reasoning, working memory, and processing speed. Seven subscales of image completion, image order, arithmetic, cubes, digit memory, and digit width were selected from 11 participants in the Wechsler test. The subscales were designed in such a way that culture and family did not influence these scales. Executive function, being an advanced subset of cognitive function, is indeed complex. Our rationale for studying both cognitive function and executive function side by side is to provide a comprehensive understanding of the potential impact of the intervention. Executive functions include basic cognitive processes, such as attentional control, cognitive inhibition, inhibitory control, and working memory as assessed through a BRIEF parental questionnaire. The BRIEF questionnaire developed for parents and teachers contains 86 items that address eight subscales of inhibition, attention shift, emotion control, initiation, working memory, strategic planning, organization, and supervision. The validity and reliability of the Persian version of these questionnaires have been confirmed in the context of Iran [41, 42].

### Statistical analysis

The sample size was calculated with 95% confidence and 80% power based on previous studies as follows:

$$n^{\prime } = \frac{{NZ^{2} P\left( {1 - P} \right)}}{{d^{2} \left( {N - 1} \right) + Z^{2} P\left( {1 - P} \right)}}$$

where $n^{\prime }$ = sample size with finite population correction,

*N* = Populations size,

*Z* = Z statistic for a level of confidence,

*P* = Expected proportion (in proportion of one), and

*d* = Precision (in proportion of one).

The sample size was calculated based on previous study [37] as a total of 90 people, with 45 people in both the intervention and control groups.

Statistical analyses were carried out using STATA software version 14.2. The baseline characteristics of the groups were examined using independent student *t* tests and chi-squared tests for continuous and categorical variables. We assessed changes between intervention groups (weeks 0–8) using a mixed-effects linear model with treatment between-subject factors (significance level; *P* < 0.05). Changes over time (0–8 weeks) between intervention groups were assessed using a linear mixed effects model with treatment as a between-subjects factor and time as a repeated measure. Age and BMI were controlled in mixed models if necessary.

This study used the linear mixed effects model test to examine anthropometric variables, food intake, and brain function. The present study involved two groups, and the study period was eight weeks, so the mixed model in three stages was used.

We first examined the differences in variables between the two intervention groups and the control group. Second, we examined the differences in variables between the two groups from week 0 to week 8. The third stage examined the differences between the two groups from week 0 to week 8. These three stages are reported as Group and Time and Group × Time in Tables 2, 3, and 4. The variables of age and BMI were adjusted in the mixed model analysis to eliminate potential confounding factors to ensure that nuts primarily caused the differences between the two groups.

**Table 2:** Anthropometric characteristics and physical activity level of participants

Variable	Nuts (N = 40)		Control (N = 40)		*β* (95% CI)	*β* (95% CI)	*β* (95% CI)
	Pre-Treatment (Mean ± SD)	Post-Treatment (Mean ± SD)	Pre-Treatment (Mean ± SD)	Post-Treatment (Mean ± SD)	Group	Time	Group × Time
Weight (kg)	48.2 ± 8.9	50.11 ± 8.9	47.04 ± 9.6	48.7 ± 10.01	1.2(− 0.3–2.7)	1.7(0.1–3.2)*	0.9(− 2.1–2.2)
Height (cm)	140.5 ± 8.2	142.4 ± 8.6	139.3 ± 7.7	141.1 ± 8.1	1.2(− 0.6–3)	1.7(− 0.08–3.5)	0.1(− 2.4–2.6)
BMI (kg/m^2^)	24.2 ± 2.5	24.2 ± 2.5	24 ± 2.5	24.1 ± 2.6	0.2(− 0.1–0.6)	0.2(− 0.1–0.5)	0.01(− 0.5–0.54)
Body fat percentage (%)	30 ± 3.8	30.2 ± 3.4	30 ± 3.7	30.1 ± 3.6	− 0.2(− 1.1–0.7)	0.1(− 0.8–1)	0.3(− 0.1–1.6)
FMI (kg/m^2^)	7.2 ± 1.5	7.4 ± 1.5	7.1 ± 1.7	7.3 ± 1.7	0.1(− 0.2–0.4)	0.2(− 1.1–0.5)	− 0.06(− 0.6–0.4)
Physical activity level (score)	31.1 ± 4	31.3 ± 3.9	31.1 ± 3.2	31.1 ± 3.2	0.02(− 1.2–1.2)	0.01(− 1.2–1.2)	0.2(− 1.5–1.9)

*BMI*  Body mass index,* FMI*  Fat mass index

Data are reported as mean ± standard deviation or percentage as appropriate. Estimated Marginal Mean ± SD presented from *T* test and *β* presented from Linear Mixed Model, at baseline (pre-treatment) and 8 weeks (post-treatment). Statistical significance *P* < 0.05. *P < 0.05 and **P < 0.001

**Table 3:** Cognitive performance of participants based on Wechsler index

Variable	Nuts (N = 40)		Control (N = 40)		*β* (95% CI)	*β* (95% CI)	*β* (95% CI)
	Pre-treatment (Mean ± SD)	Post-treatment (Mean ± SD)	Pre-treatment (Mean ± SD)	Post-treatment (Mean ± SD)	Group	Time	Group × Time
Total Wechsler score	119 ± 22.8	161.1 ± 22	118.1 ± 23.8	137.3 ± 26.3	0.7 (− 3.2, 4.6)	19.15 (15.1, 23.1) **	23.1 (17.5, 28.7) **
Picture completion	17 ± 2.9	20 ± 19.5	17.45 ± 2.9	19 ± 2.7	− 0.4 (− 1.4, 0.5)	1.4 (0.5, 2.4) **	2.07 (0.7, 3.4) **
Picture concept	22.4 ± 7.3	33 ± 7	22 ± 7.6	26 ± 7.2	0.5 (− 1.6, 2.7)	4.05 (1.8,6.2) **	6.3 (3.2, 9.5) **
Block design	20.2 ± 9.1	30.5 ± 8.5	19.1 ± 7.6	23.3 ± 8.5	1.1 (− 1–3, 3)	4.2 (2.04, 6.4) **	6.02 (2.9, 9.1) **
Object assembly	16 ± 6.5	23.1 ± 3.4	16 ± 5.1	19.2 ± 4.9	− 0.2 (− 2.1, 4)	3.5 (1.7, 5.2) **	3.8 (1.4, 6.3) **
Short − term memory	8.2 ± 2.5	11.3 ± 2.6	8 ± 2.04	9 ± 1.5	0.3 (− 0.5, 1.1)	1.07 (0.1, 1.9) *	2.02 (0.7, 3.2) **
Digit span	28.1 ± 5.6	36 ± 7.1	29.1 ± 7.9	34 ± 7.9	− 0.9 (− 2.9, 1.09)	4.7 (2.7,6.7) **	2.8 (0.02, 5.7) *
Arithmetic	7 ± 0	7 ± 0	7 ± 0	7 ± 0			

Data are reported as mean ± standard deviation or percentage as appropriate. Estimated Marginal Means ± SD presented from *T* test and *β* presented from Linear Mixed Model, at baseline (pre-treatment) and 8 weeks (post-treatment). Statistical significance *P* < 0.05. *P < 0.05 and **P < 0.001

**Table 4:** Executive performance of participants based on BRIEF index

Variable	Nuts (N = 40)		Control (N = 40)		*β*(95% CI)	*β*(95% CI)	*β*(95% CI)
	Pre-treatment (Mean ± SD)	Post-treatment (Mean ± SD)	Pre-treatment (Mean ± SD)	Post-treatment (Mean ± SD)	Group	Time	Group × Time
Total BRIEF score	62 ± 28.4	49.1 ± 24.3	61 ± 22.7	65 ± 25.2	0.85(− 8.3,10)	4(− 5.1,13.1)	− 16.5(− 29.4, − 3.5)*
Inhibition	11 ± 6.5	7.6 ± 4.3	9.1 ± 4.6	9.2 ± 5.3	1.6(− 0.4,3.7)	0.07(− 2,2.1)	− 3.2(− 6.1, − 0.3)*
displacement	9 ± 4.3	6.4 ± 4	8.2 ± 3.2	8.5 ± 4.7	0.5(− 1.1,2.1)	0.3(− 1.3,1.9)	− 2.6(− 5, − 0.2)*
Emotion control	10 ± 4.7	8.3 ± 4.6	9.5 ± 4.5	10 ± 5.1	0.3(− 1.4,2.1)	0.1(− 1.5,1.9)	− 1.7(− 4.2,0.7)
Initiation	6.4 ± 3.1	5.3 ± 2.8	7 ± 3.5	8 ± 3.5	− 0.6(− 1.8,0.5)	0.9(− 0.2,2)	− 2.05(− 3.7, − 0.3)*
Working memory	7.1 ± 3.7	6 ± 3.6	7.1 ± 3.1	7 ± 3.5	0.05(− 1.3,1.4)	− 0.1(− 1.5,1.2)	− 1.3(− 3.3,0.6)
Planning	6.2 ± 6	8 ± 4.7	9.3 ± 5	9 ± 4.8	− 0.1(− 2,1.8)	− 0.4(− 2.4,1.5)	− 1.1(− 3.8,1.6)
Organization	6.3 ± 3.9	5 ± 3.5	7 ± 3.7	8 ± 4.3	− 0.3(− 1.7,1)	1.07(− 0.3,2.4)	− 2.5(− 4.5, − 0.5)*
Monitoring	7 ± 3.7	6 ± 3.3	7 ± 3.5	7.3 ± 3.4	− 0.1(− 1.5,1.2)	0.6(− 0.7,1.9)	− 1.6(− 3.5,0.3)

Data are reported as mean ± standard deviation or percentage as appropriate. Estimated Marginal Mean ± SD presented from *T* test and *β* presented from Linear Mixed Model, at baseline (pre-treatment) and 8 weeks (post-treatment). Statistical significance *P* < 0.05. *P < 0.05 and **P < 0.001

## Results

**Fig. 1:**
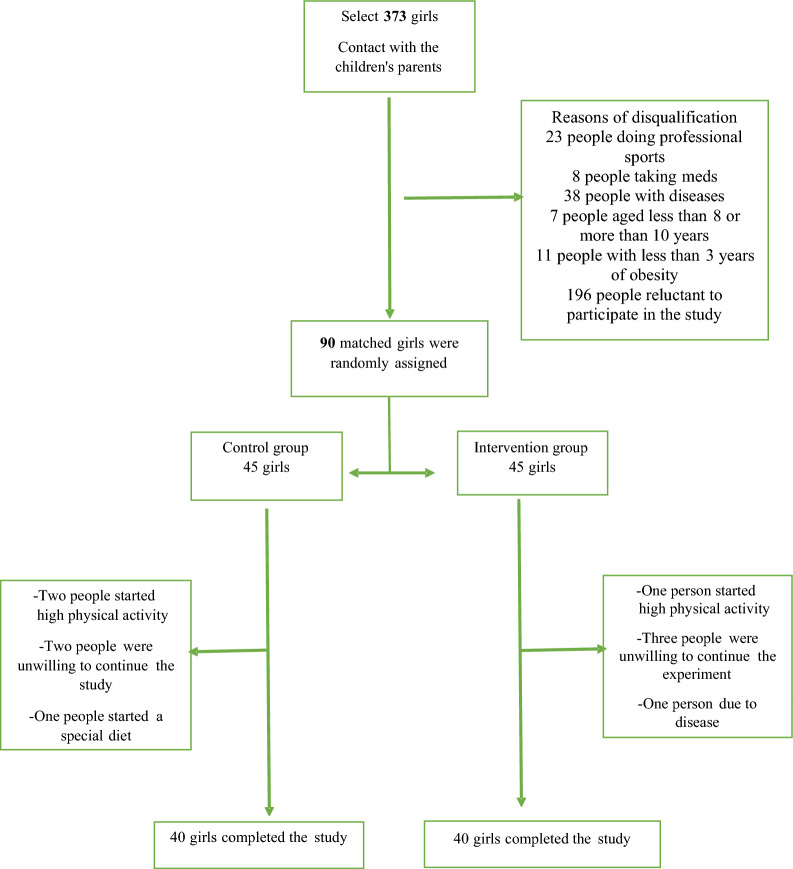
Consort diagram for participant assignment through the study

In this clinical trial study, 90 qualified obese girls aged 8–10 were enrolled. During the study five girls in each group were excluded and finally 40 obese girls in each group completed the study (Fig. 1). Demographic information is shown in Table 1 and there were no significant differences between two groups in demographic data. The mean age was 9 ± 0.7 years. Moreover, dietary intake is demonstrated in Supplementary Table 2. At the beginning of the study, there was no significant difference in food intake between the groups.

Table 2 shows anthropometric and physical activity information. No statistically significant differences were observed for weight (kg), height (cm), BMI (kg/m^2^), body fat percentage (%), FMI (kg/m^2^), physical activity level (score) in intervention groups versus control group (*P* > 0.05).

Table 3 displays the data of cognitive performance outcomes based on Wechsler test. The intervention group showed a significant increase in total Wechsler score (differences: 23.1, 95% CI: 17.5, 28.7; *P*_Time×Group_ < 0.001) compared to control group. Moreover, we assessed all seven domains of Wechsler test and analysis showed that picture completion (*P*_Time×Group_ < 0.001), picture concept (*P*_Time×Group_ < 0.001), block design (*P*_Time×Group_ < 0.001), object assembly (*P*_Time×Group_ < 0.001), short-term memory (*P*_Time×Group_ < 0.001), and digit span (*P*_Time×Group_ < 0.05) were significantly improved in nuts group compared to the control group after the 8th weeks. However, there was no significant difference in the test scores for arithmetic.

Table 4 shows the executive performance data based on the BRIEF test. In this test, the decrease in score shows the improvement of executive performance. There was a significant improvement of the total BRIEF score in intervention group versus control group (differences: − 16.5, 95% CI: − 29.4, − 3.5; *P*_Time×Group_ < 0.05) after eight weeks. Furthermore, a significant improvement in the BRIEF score domains including inhibition (*P*_Time×Group_ < 0.05), displacement (*P*_Time×Group_ < 0.05), initiation (*P*_Time×Group_ < 0.05), and organization (*P*_Time×Group_ < 0.05) were observed in the intervention group. However, the effect of nuts intervention on emotion control, working memory, planning, and monitoring were not significant.

## Discussion

This is the first study to investigate the effect of nuts on improving cognitive and executive function in children. Consumption of nuts for 8 weeks improved the overall score of cognitive function and executive function in children with obesity. Also, short-term consumption of nuts caused significant changes and improvements in short-term memory, digit span, picture completion, picture concept, block design, initiation, displacement, inhibition and organization in obese children.

In this study, obese children were chosen because other studies had shown the relationship between childhood obesity and reduced cognitive function and poor academic skills [7–9]. However, these studies have indicated the need for more detailed investigations on the subscales of these variables [14, 34]. Additionally, the logic for selecting the age group of children is that they have more neuroplasticity at young ages and improving cognitive function at this time can have better effects on brain function in the future, and prevent deterioration and cognitive decline [34, 36]. Studies have shown that cognitive dysfunction decreases quality of life and academic performance [9, 10, 43]. Obesity impairs cognitive function through brain tissue issues, affecting food regulation. Children with lower executive functions are more sedentary and snack more, with higher BMI linked to poorer executive functions. Preventing obesity during brain development is crucial for executive functions [6–8, 44, 45].

Consumption of nuts, especially walnuts, almonds, and hazelnuts, can improve the quality of the diet and enrich the children’s macronutrients and micronutrients status [31, 32], and better cognitive function is associated with a higher-quality diet [46]. In line with our finding, prospective studies have reported that long-term, high-dose consumption of nuts is associated with better cognitive function [26, 47, 48]. Moreover, in the trial study conducted by Martinez et al. [38] showed that 6.5 years of intervention of 30 g of nuts (walnuts 15 g, almonds 7.5 g, and hazelnuts 7.5 g) accompanied by the Mediterranean diet improved cognitive function in old ages. In another RCT, consumption of 3 oz/d almond for six months could improve the cognitive function of middle-aged people, but lower and duration of less than six months, no significant effect on cognitive function was observed [49]. However, 12 weeks of almond intervention on overweight and obese adults aged 50–80 years had no significant effect on improving the subjects’ cognitive function [30]. Our results verify these findings, suggesting that the duration and dosage of nut consumption play critical roles in cognitive health benefits. Another study on healthy older adults indicated that walnut consumption for two years, of 15% of energy intake (30–60 g), did not alter cognitive function, although functional brain MRI results showed that walnut consumption might lead to cognitive decline delay [14]. This emphasizes the potential of nuts to contribute to cognitive health through mechanisms that include improved diet quality, enriched macronutrient and micronutrient status, and their associated effects on brain function.

The results of lipid profile analysis and the amount of protein in Iranian nuts used in the study of heat showed significant amounts of oleic acid, linoleic acid, and protein. Walnuts, almonds, and hazelnuts also contain bioactive compounds, which can protect neurons. The human brain has a high percentage of lipids and unsaturated fatty acids. These three nuts have excellent lipid profiles and are rich in MUFA and PUFA, such as oleic acid, linoleic acid, and linolenic acid. The nuts are also rich sources of vitamins E and B, phytochemicals, polyphenols, essential amino acids such as arginine, glycine, glutamic acid, histidine, as well as micronutrients like magnesium, selenium, copper, iron, and zinc, all having beneficial effects on brain functions [50]. Generally, the mentioned nuts increase antioxidant capacity, and reduce oxidative stress, free radicals, oxidation, lipid peroxidation, inflammation, A*β* induction, and A*β* oligomers [51–53]. In addition to improving blood flow and protection against arterial plaque formation [12, 54], they can also enhance brain function and prevent brain damage. Research have also presented that nut contain macronutrients and micronutrients that include synergistic effects on each other, protecting neurons from destructive agents, enhancing blood flow to the brain, improving cognitive function, and preventing brain damage in obese children.

### Strengths of the present study

First, we used a combination of nuts (walnut, almond, and hazelnut). Second, two tests were applied to assess cognitive function (completion of one test by children and another test by parents). Third, possible confounder factors such as lifestyle, food intake, and socioeconomic status of individuals were controlled.

### Limitations and suggestions

In this study, due to the onset of the Covid-19 pandemic and global quarantine, evaluation was not possible at week 16. Also, we had financial limitations. We recognized that learning content (e.g., math, sports) might have influenced cognitive and executive functions. Despite our efforts to control these variables, they could have impacted outcomes. The improvements may not be solely due to nut consumption, highlighting the need for further research to isolate the intervention’s specific effects. It is also suggested that in future studies, the mood status of the participants in the study be examined. Due to the sensitivity of parents and budget constraints, it was impossible to check blood factors and inflammatory indicators. It is recommended to perform research on both sexes by examining inflammatory and oxidative markers. It was not possible to blind participants or staff in the current RCT blinding study due to nutritional interventions. Nonetheless, the group assignment was concealed from the outcome assessor. Finally, further dose–response studies on this subject are suggested.

## Conclusions

Over a period of 8 weeks, incorporating nuts into the diets of obese children resulted in an increase in their cognitive and executive function scores. Furthermore, consuming nuts for a short period of time led to significant enhancements in various areas such as short-term memory, digit span, picture completion, picture concept, block design, initiation, displacement, inhibition and organization among children with obesity.

## Supplementary Information

Supplementary file 1

## Data Availability

The data that support the findings of this study are available from the corresponding author, upon reasonable request.
