# Supplementary material for: The effects of nuts intake on cognitive and executive function in obese children: a randomized clinical trial
Source: J Health Popul Nutr. 2025 Mar 12;44:74. doi: 10.1186/s41043-025-00804-7 (PMC11899795; doi:10.1186/s41043-025-00804-7)
Supplement: Supplementary file 1 [file 41043_2025_804_MOESM1_ESM.docx]

**Supplemental Table 1:** [Protein and fatty acid composition of nuts](https://pubmed.ncbi.nlm.nih.gov/17125530/)

| In 30 gr | Hazelnut% | Almond% | Walnut% |  |
| --- | --- | --- | --- | --- |
| 4.68 | 13.67 | 20.43 | 14.21 | **Protein** |
| 0.0075 | 0.02 | 0.05 | 0.02 | **C14** |
| 1.869 | 5.82 | 6.39 | 6.46 | **C16** |
| 0.077 | 0.2 | 0.78 | 0.08 | **C16:1** |
| 0.0125 | 0.03 | 0.05 | 0.05 | **C17** |
| 0.057 | 0.07 | 0.1 | 0.03 | **C17:1** |
| 0.734 | 2.2 | 1.72 | 2.96 | **C18** |
| 14.936 | 79.81 | 73.02 | 23.24 | **C18:1 Cis** |
| 0.0035 | 0.02 | 0.03 | - | **C18:1 Trans** |
| 10.32 | 11.53 | 17.75 | 54.21 | **C18:2 Cis** |
| 0.006 | - | - | 0.04 | **C18:2 Trans** |
| 1.993 | 0.09 | 0.04 | 12.87 | **C18:3 Cis** |
| 0.0075 | 0.1 | - | - | **C18:3 Trans** |
| 0.0225 | 0.11 | 0.02 | 0.09 | **C20** |
| 0.006 | - | - | 0.04 | **C20:1** |

*C4, C8, C10, C12 and C14:1 fatty acid not detected

**Supplemental Table 2:** Dietary intake in two groups at the baseline and end of the study.

| Variable | Nuts  (N=40) | | Control  (N=40) | | β(95% CI ) | β(95% CI ) | β(95% CI ) |
| --- | --- | --- | --- | --- | --- | --- | --- |
|  | Pre-Treatment  (Mean ± SD) | Post-Treatment  (Mean ± SD) | Pre-Treatment  (Mean ± SD) | Post-Treatment  (Mean ± SD) | Group | Time | Group × Time |
| Energy (Kcal) | 1832±148.4 | 1847±136.6 | 1835±146.6 | 1878±152.3 | -2.9(-37.9-32.1) | 42.6(7.5-77.6)* | -28.7(-78.3-20.7) |
| Carbohydrate (percent of calorie) | 47±4 | 44.8±3.4 | 45.5±3.8 | 45.1±3.8 | 1.2(-0.4-2.8) | -0.4(-2-1.2) | -1.5(-3.8-0.7) |
| Protein (percent of calorie) | 13±1.6 | 14±1.6 | 13.4±1.5 | 13.3±1.9 | -0.4(-1.2-0.2) | -0.07(-0.8-0.6) | 1(-0.002-2)* |
| Fat (percent of calorie) | 40.1±3.9 | 41.1±3.3 | 41±3.5 | 41.4±3.4 | -0.7(-2.2-0.7) | 0.5(-0.9-2) | 0.4(-1.6-2.5) |

Data are reported as mean ± standard deviation or percentage as appropriate.

Estimated Marginal Means ± SD presented from T-test and β presented from Linear Mixed Model, at baseline (pre-treatment) and 8 weeks (post-treatment).

Statistical significance P < 0.05. * P < 0.05 and **P < 0.001
